# Scientific Study of *Gentiana kurroo* Royle

**DOI:** 10.3390/medicines4040074

**Published:** 2017-10-12

**Authors:** Bhat Mohd Skinder, Bashir Ahmad Ganai, Abdul Hamid Wani

**Affiliations:** 1 Centre of Research for Development (CORD)/Department of Environmental Science, University of Kashmir Srinagar, Srinagar 190006, Jammu and Kashmir (J&K), India; bbcganai@gmail.com; 2 Department of Botany, University of Kashmir Srinagar, Srinagar 190006, Jammu and Kashmir (J&K), India; ahamidwani@yahoo.com

**Keywords:** *Gentiana kurroo* Royle, medicinal plant, phytoconstituent, antimicrobial, anti-arthritic, analgesic, anti-diabetic, bioactive molecule and bio-prospecting

## Abstract

The present investigation was carried out to review and highlight the potential phytochemicals and medicinal phenomena of the critically endangered medicinal plant, *Gentiana kurroo* Royle of the western and north-western Himalayas. The medicinal plant is heavily exploited for root and rhizome. Due to its endemic nature and the high rate of exploitation from its natural habitat, this species had become critically endangered. The phytochemical screening of the plant revealed that the plant contains some vital phyto-constituents (iridoids, xanthones, C-glucoxanthone mangiferin, and C-glucoflavones) that have a medicinal value for various acute and chronic diseases. Several researchers have carried out experimental work to validate the folkloric use of the medicinal plant for different ailments like antibacterial, antioxidant, anti-arthritic, anti-inflammatory, analgesic activities and anti-diabetic activity. However, it is yet to be confirmed the antifungal activity of the same plant. Because of endemic nature and high rate of exploitation there is need for alternative method called bio-prospecting of Endophytes from the plant, to carry out the production and characterization of bioactive metabolites for pharmacological uses and can become a conservative tool for the medicinal plant.

## 1. Introduction 

**Figure 1:**
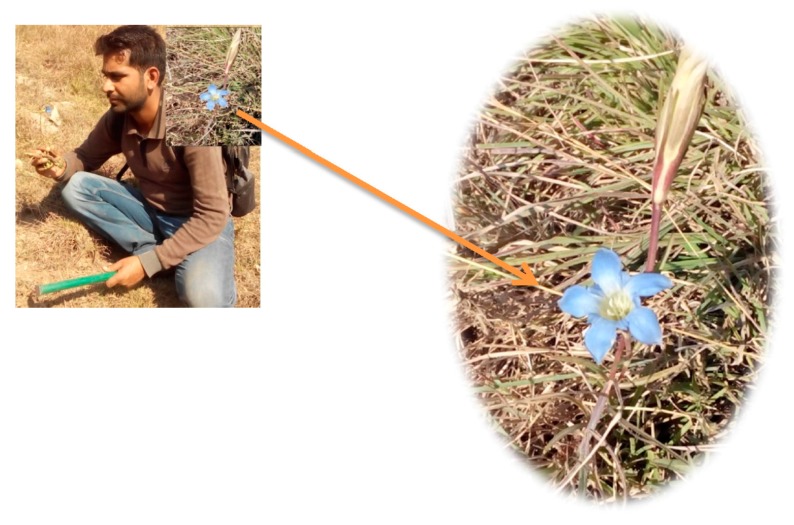
*G. kurroo* Royle collected from the Khrew village in the district of Pulwama, J&K.

The common name of Gentiana has been derived from “Gentius”, a king of Illyria (Europe), who is believed to have discovered the medicinal value of the Gentian root. In fact, the specific name of *G. kurroo* Royle (Figure 1) is from the local name for the root of the plant, “Karu” meaning bitter. *G. kurroo* Royle commonly known as “Indian Gentian” in English or Himalayan Gentian, “Karu” in Hindi, “Traayamaana” in Sanskrit [1,2,3], however in Kashmir Himalaya it is called as “Nilkanth” but Bakarwals (high-altitude goatherds/shepherds) living in the area of Sinthon top (asl-3800 m) and Daksum (asl-2438 m), named it “Tazakhzand” in their local language. *G. kurroo* Royle is rosette-forming perennial herb and a critically endangered medicinal plant [4]. 

## 2. Habitat and Distribution of *G. kurroo* Royle

*G. kurroo* Royle belongs to the family Gentianaceae (family of flowering plants). The Gentianaceae family is represented by more than 90 genera and 1650 species; they are annual and perennial herbs or shrubs, native to northern temperate areas of the world. Nearly 360 species of genus *Gentiana* have been recorded [5,6,7,8], 62 species with 16 genera out of the total number are observed in temperate regions of India [9,10]. The species is distributed in the Himalayan region across India, Pakistan and Nepal [11]. It is endemic to the north-western Himalayas [12] and commonly grows in Kashmir, Himachal Pradesh, and adjoining hills of the north-western Himalayas at an altitude of 1500–3000 m asl [8,10,13,14] In Kashmir, it is usually found on south facing steeper slopes along dry and rocky sloppy grasslands and sparsely shrubby scrubs [15], whereas, in Himachal Pradesh (1700–2000 m), it has become intermittent in subalpine to alpine meadows [10,16]. More than 80% of the population decline of the species has taken place in India in a time period of ten years [17,18]. Therefore, the species is assessed as critically endangered [19]. India has the majority of the geographical range (80%) and therefore, situation in India is considered as the representative of the global population of the species.

The Gentiana family is characterized by six genera and 55 species in the Kashmir Himalaya, an important domain of the Himalaya hotspot [20]. The genus Gentiana has high diversity in this province with 35 species. Out of these species, 31 reach alpine or sub-alpine levels [21]. *G. kurroo* Royle was described by [22] as a new plant species on the basis of specimens collected from the areas of the north-western Himalayas (Mussooree, Kuerkoolee, Budraj and Shimla). *G. kurroo* was first reported in the Kashmir Himalaya by [23] at an altitude of 1850–2000 m (a.s.1) from the Pahalgam area, followed by [24] from the localities of Kangan and Wangat. In many floristic works dealing with Kashmir Himalaya, the plant species has been included, not based on the author’s personal collections but by citing the pre-1943 collections ([21,25,26,27]. Due to the large scale exploitation the plant extinct from the Dachigam National Park, however, *G. kurroo* Royle has been rediscovered in November, 2004, on the floristic expedition to the Dachigam National Park in Kashmir Himalaya after more than sixty years since it was reported earlier [14]. *G. kurroo* Royle known to be adulterated/substituted with roots of *Gentiana tenella, Picrorrhiza kurroa*, *Gentiana decumbens*, *Exacum bicolor* [28,29,30,31] for its high demand in international market and limited supply from natural systems. However, the purity and authenticity of crude drugs can be performed by macroscopic, microscopic and anatomical observation besides chemical and ash analysis [32,33,34].

## 3. Life Cycle of *G. kurroo* Royle

*G. kurroo* shows propagation through rhizome cuttings, seeds, somatic embryogenesis and micro proliferation of shoot nodal segments. The shoot system of the *G. kurroo* is represented by flowering branches only with culine leaves. The stem is a modified rhizome whereas the root system consists of the rhizome and adventitious root. Flowering starts from the third week of August to first week of November with the peak from 15 September to 20 October. On an average a plant produces 20 flowers, and the ideal time for seed harvest is the first fortnight of November [10,35].

## 4. Rationale of the Study

Traditionally, a number of plants and their preparations have been in use for the treatment of different diseases. Awareness of plant-based medications and therapeutics are continuously increasing worldwide, hence the recognition and demand. However, very few of these have been validated scientifically through rigorous in vivo animal studies and clinical trials. Most of the available scientific data confirming the disease curing potential of traditionally used plants but lacks systematic studies on their mode of action, efficacy, stability, toxicity and safety. In-depth scientific validation studies are required to validate the traditional medications as alternative and complementary drugs for the treatment of various diseases.

Although little work has been carried out regarding *G. kurroo* Royle, maximum efforts have still been put in to compile all the research work in the present comprehensive review. The experimental work carried out by scientists to investigate the promising phytochemicals and to validate the folkloric use of *G. kurroo* Royle through different activities; for example, antimicrobial, antioxidant, anti-arthritic, anti-inflammatory, analgesic and anti-diabetic activities have been highlighted in this review paper.

## 5. Phytochemistry

**Figure 2:**
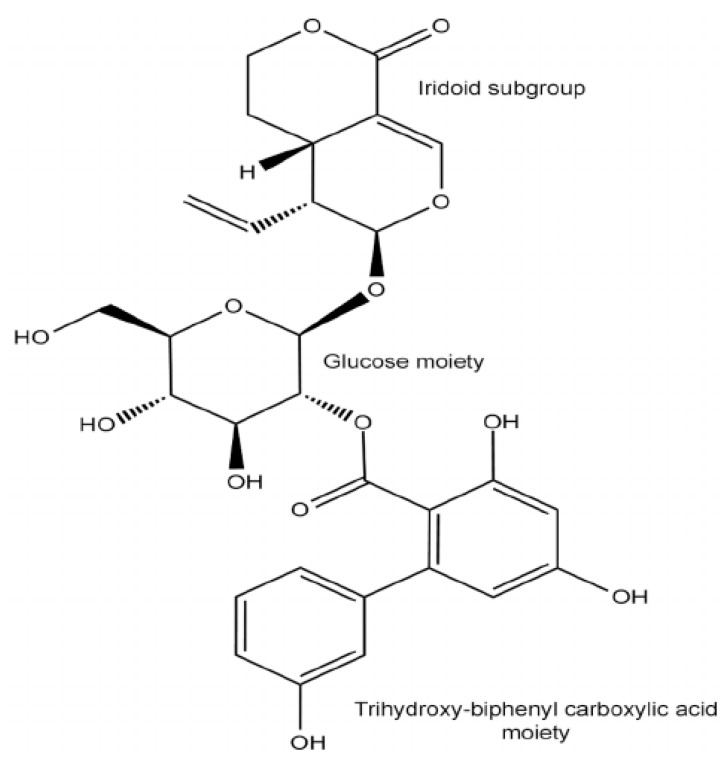
Structure of Amarogentin—a secoiridoid glycoside. Amarogentin consists of three essential subgroups, the iridoid group, the glucose moiety and the biphenyl-triol rings (Source [44,45]).

The family Gentianaceae have taxonomically useful types of compounds and pharmacological actions. The various compounds like iridoids, xanthones, C-glucoxanthone mangiferin, and C-glucoflavones have been recorded. The iridoids (mostly secoiridoid glucosides) appear to be present in all species studied [36], whereas 90 different compounds of iridoids have been reported from 127 species in 24 genera. Although Xanthones are not commonly present in Gentianaceae, about 100 different compounds have been reported from 121 species in 21 genera. However, the C-glucoxanthone mangiferin has a more limited distribution than the iridoids and the normal xanthones, although it has been reported from 42 species in seven genera. Similarly, only nine different compounds of C-glucoflavones have been reported so far from a total of 78 species in bube genera and are much less variable than the iridoids and the xanthones [37]. The family contains most bitter compounds; even at a dilution of 1:58,000,000, one tastes bitter, known as Amarogentin (Chirantin) (Figure 2), a glycoside, and is used as a scientific basis for measuring bitterness [38,39,40,41]. Bitter products have been traditional remedies for loss of appetite and fever and are still included in many “tonic” preparations [42,43]. In the present case, it is not possible to document the phytochemistry of all species of the family Gentianaceae; therefore only one species (*G. kurroo* Royle) of genus Gentiana will be taken into detailed consideration because of its nature as a critically endangered species in the Kashmir Himalayas, as it is mostly extracted for the different ailments of human diseases.

The phytochemical screening of *G. kurroo* Royle revealed various vital phytoconstituents as depicted in Table 1. The quantitative estimation (%) of chemical constituents and fraction of flavonoids of flower tops of *G. kurroo* Royle has been depicted in Table 2, whereas the total flavonoid and phenolic content of the leaf and root extract are given in Table 3. However, besides flavonoid and phenolic content, there are other constituents present in the root extract, as depicted in Table 4. The critically endangered drug herb is mostly extracted from its natural habitat for root and rhizome. The roots and rhizomes of *G. kurroo* Royle have been recorded in the Indian pharmaceutical codex [8]. The root and rhizome are source of Iridoid glycosides-gentiopicrine, gentiamarin, amaroswerin, and the alkaloid gentianine [46,47,48]. The dried roots contain 20% of a yellow, transparent, and brittle resin [23,29,48], aucubin, catalpol, 6-*O*-vanilloyl catalpol, 6-*O*-cinnamoyl catalpol, [49,50]. However, the leaves also contain some of the important bitter compounds; noticeably moreso than the roots [51]. Leaves contain iridoid glycoside 2′-(2,3-dihydroxybezoyloxy)-7-ketologanin [52] and about 16 volatile aroma compounds [53]. Some of the principal components in the leaf extracts of *G. kurroo* Royle are dimethyl sulphide (14.7%), 2-ethylfuran (17.5%), 1,8-cincole (7.8%), a-terpinyl acetate (23.5%) and methandriol (12.6%). The other chemical components with lesser percentage are 1,3-propanediol (2.1%), 2-methyl sulphide (2.1%), 3-methyl butanol (4.4%), pentanal (3.2%), hexanal (2.7%) and 7-oxabicyclo (4,1,0)-heptane (2.0%) ([53]). Some other components isolated are morroniside and gentiopicroside [54].

## 6. Ethno-Pharmacology

*G. kurroo* Royle has been found to have enormous medicinal properties reported by several researchers. The medicinal values date back to when human beings first got to know natural cures for different diseases from natural products. In folkloric treatment, leaf powder of *G. kurroo* (Neilkanth) is mixed with oil and is applied on ulcer and fungal infection [66]. However, the root of *G. kurroo* is used in stomach-ache and in urinary infections [67]; the root with ginger root powder is also used for curing high fevers [68]. The roots were also used as bitter tonic, and as an antiperiodic, expectorant, astringent, stomachic, anti-inflammatory, antipsychotic, sedative, anthelmintic and antibacterial [69]. Gentianine (alkaloid) in *G. kurroo* possesses anti-infammatory, analgesic, anticonvulsant, hypotensive, antipsychotic, sedative, diuretic, antimalarial, anti-amoebic and antibacterial properties and Amaroswerin acts as gastro-protective [48], whereas some traditional doctors use the whole plant against cough, fever, headache, liver ailments and as a blood purifier [70]. The drug obtained from *G. kurroo* is very helpful in removing all kinds of weakness and overtiredness of body from prolonged illness, recovers digestive system and lack of appetite [71]. In the Ayurvedic (Unani) system of medicine, the flower tops (Gule-Ghafis) are used for treatment of inflammation, pain, antipyretic and hepatitis [61,62] and in the preparation of tonics for stomachic [72]. It is also curative for the skin disease leucoderma, leprosy, dyspepsia, colic, anorexia, flatulence, helminthiosis, anti-inflammatory, amenorrhea, dysmenorrhoeal, haemorrhoids, strangury, constipation, urinary infections as an antiseptic, bitter tonic, cholagogue and bronchial asthma [46,73,74]. However, there are some scientific validations of the folkloric uses of the critically endangered medicinal plant *G. kurroo* Royle.

## 7. Antibacterial Activity

The recent study has revealed the antibacterial and antioxidant activity of the extracts of *Gentiana kurroo*, as the extracts prevented the growth of both Gram positive and Gram negative bacteria. The extracts of roots and leaves of *G. kurroo* possessed relatively higher antibacterial activity against Gram positive bacteria than the Gram negative. The possible reason for antibacterial activity is due to high content of flavonoids, involved in the inhibition of nucleic acid biosynthesis and metabolic processes [75,76,77]. The antibacterial activity of root extract was found to be comparatively higher than that of leaf extract and it was highest against *Micrococcus luteus* (0.15 mg/mL) and lowest activity against *Salmonella enteritidis* (0.75 ± 0.05). Leaf extract also exhibited a similar trend as shown in Table 5, whereas the minimal inhibitory concentration (MIC) values were higher than those of the root extract [59]. However, the study does not find out the peculiar bioactive molecule that specifically regulates the growth of bacteria, but has successfully proved that *G. kurroo* could be a potential source of broad spectrum antibacterial agents and can be used as preservatives in food and non-food systems; thus, further phytochemical analysis is required for the isolation of bioactive molecules from the plant that may show a broad spectrum of pharmacological activities.

## 8. Antioxidant Activity

**Figure 3:**
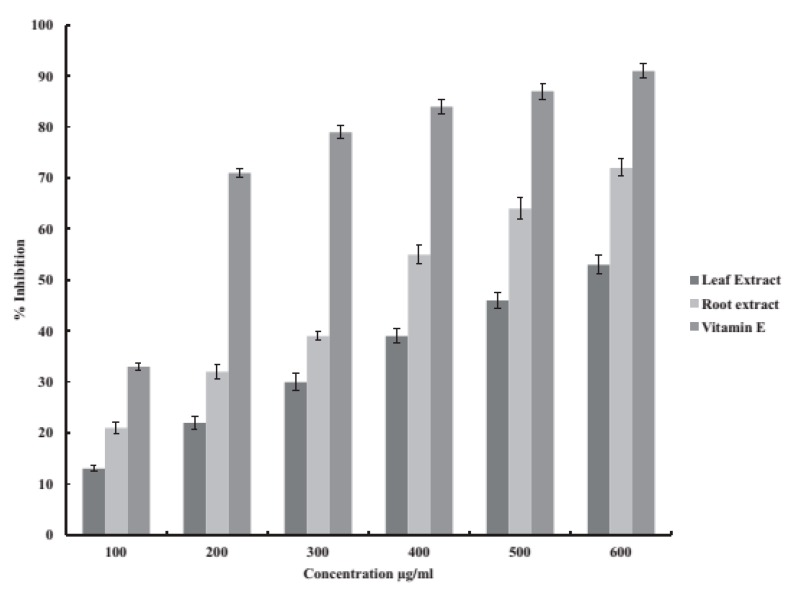
Free radical scavenging activity of methanolic extracts of leaves and roots of *G. kurroo* Royle (Source: [59]).

**Figure 4:**
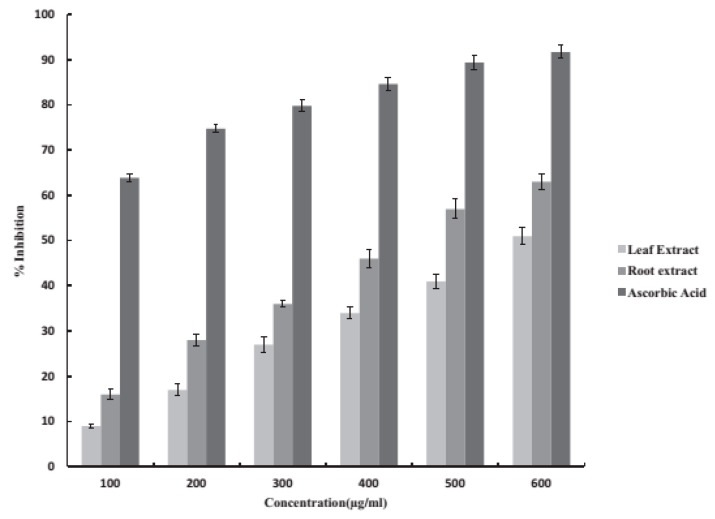
Superoxide scavenging activity of methnolic extracts of leaves and roots of *G. kurroo* Royle (Source: [59]).

The methanolic extracts of leaves and roots of *G. kurroo* showed high phenolic and flavonoid content. Phenolic compounds are important plant constituents for their free radical scavenging ability, enabled by their hydroxyl groups, and the total phenolic concentration might be used as a source for rapid screening of antioxidant activity [78] and are also involved in the oxidative stress tolerance of plants. Flavonoids are highly effective scavengers of most oxidizing molecules concerned with several diseases [79,80]. On the other hand, flavonoids suppress reactive oxygen formation, chelate trace elements involved in free-radical production, scavenge reactive species, up-regulate and protect antioxidant defences [81]. The methanolic extracts of root as compared to the methanolic extract of leaves showed comparatively high antioxidant activity, which could be related to the total flavonoid and phenolic content of the two extracts [59,80] (Table 6) (Figure 3 and Figure 4).

## 9. Anti-Arthritic and Anti-Inflammatory Activity

The recent study [84] and [60] has shown positive results from the extracts of *G. kurroo* regarding acute and chronic anti-Inflammatory test. To determine the acute inflammatory effect, rat carrageenin-induced paw edema model was used. Different plant extracts were screened for anti-inflammatory activity at a dose of 250 mg/kg body weight. The maximum potential for suppressing the inflammatory response was shown by methanolic extract of *G. kurroo.* The observed inhibitory effect in the paw edema of Wistar rats was 47.62% and it was found to be significant (*p* < 0.05) as compared to control group (55.24%). However, a dose of 750 mg/kg body weight has shown maximum activity (67.27%) which was even found to be higher than that of the standard drug (56.36%). The results were found to be statistically significant compared to control group but non-significant with standard group at *p* < 0.05.

**Figure 5:**
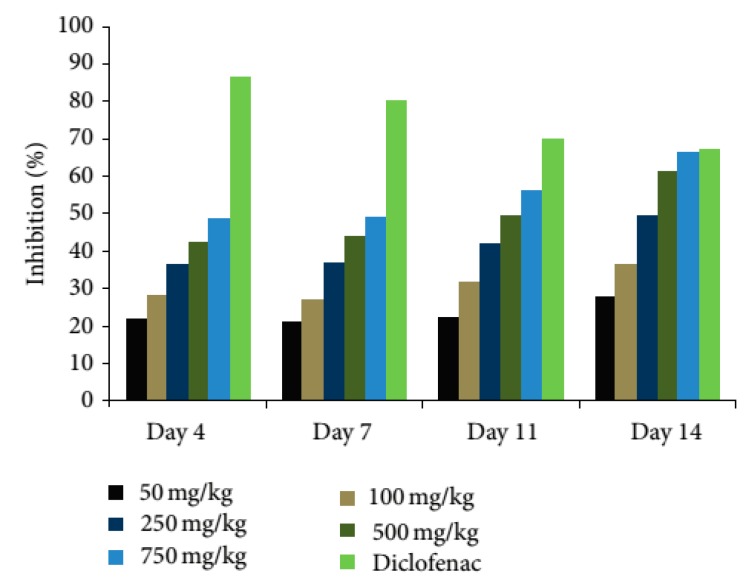
Inhibition of paw edema in adjuvant arthritis with different concentrations of methanolic extract of *G. kurroo* Royle (Dose and time dependent) (Source: [85]).

Chronic Anti-Inflammatory Test: For this study, mycobacterium-induced adjuvant arthritis as a model of chronic inflammation was used. Male Wistar rats were taken in seven groups, with each group having the same number of animals (*n* = 5). The results revealed that the methanolic extract has a dose as well as time-dependent inhibitory effect on the edema formation (Figure 5). The increased activity with increased doses may be due to high concentration of bioactive agent/s in the extract. Methanolic extract of *G. kurroo* has more pronounced effect as compared to other extracts and consequently indicates the inhibition of chemical mediators of inflammation. The results were found to be very significant as related to arthritic control at *p* < 0.05. It may be due to the incidence of more bioactive agent(s) in the extract [60,85]. Anti-inflammatory property of *G. kurroo* could be related to secondary metabolites such as terpenoids or flavonoids; monoterpenoids such as camphene, borneol, and *β*-pinene have similar properties [55,56], and flavonoids such as 6-methoxytricin show anti-inflammatory and analgesic activities [57]. This could also be because of the inhibition of proinflammatory cells [60,85]. Although the study related to the anti-inflammatory drug obtained from a methanolic extract of *G. kurroo* against rheumatoid arthritis is prolific, it has the limitation of the possible mechanism and the identification of the bioactive compound from the extract of *Gentiana kurroo*; however, it is confirmed that the *G. kurroo* serves as a drug source against inflammation and rheumatoid arthritis.

## 10. Analgesic Activity

The study being carried out by [58] on the analgesic activity of *G. kurroo* was based on two tests: an acetic acid-induced writhing test was used for detecting both central and peripheral analgesia, whereas the hot plate test is most sensitive to centrally-acting analgesics. In the acetic acid-induced writhing test, the methanolic extract of *G. kurroo* root at a dose of 250 and 500 mg/kg body weight revealed a significant (*p* < 0.05) decrease in number of writhings (63.38% and 73.70% inhibition) provoked by acetic acid in a dose-dependent manner, and the results were comparable with the standard drug diclofenac sodium (71.61% inhibition). It could be possible that extracts produced analgesic effect due to the inhibition of synthesis or action of prostaglandin [86]. However, in the case of Eddy’s hot plate test, the extract showed a significant (*p* < 0.05) rise in reaction time (increase threshold potential of pain) in a dose-dependent manner to the thermal stimulus at different time of observation (0–120 min) in comparison with control. Thus, results revealed that *G. kurroo* Royle possesses potent analgesic effect against different stimuli. The possible mechanism was found to be due to an inhibition of both peripherally and centrally-mediated nociceptive.

## 11. Anti-Diabetic Activity

The plant is also used against diabetes [87]. There is no literature available in support of the scientific validation of anti-diabetic activity of *G. kurroo* Royle; however, the recent study held in 2017 confirmed the counter-diabetic capability of *G. kuroo* Royle. The experiments carried out on rats, shown the extracts of *G. kurroo* Royle were found to improve the glycaemic control in oral glucose tolerance tests as it was observed even in normal rats; the glucose load is quickly cleared by the plant extracts. The methanolic and hydroethanolic extracts (each at the dose level of 500 mg/kg of body weight) were found to overcome the main symptoms of the diabetes, i.e., polyphagia, polydipsia and polyuria. It was also observed the weight loss to lessen in diabetic rats. It has a controlling power in hyperglycaemia and can viably work against other metabolic deviations created by diabetes in rats. The possible reason for the antidiabetic activity of *G. kurroo* Royle are due to bioactive principles like Swertiamarin, swertisin and lupeol [88]. The systematic study is required for the understanding of suitable mechanism of hypoglycaemic potential of the plant extracts of *G. kurroo* Royle.

## 12. Conservation Strategy

Traditional extraction of medicinal plants on a large scale could be minimized so as to save these from becoming critically endangered. This could be achieved through the exploration of new ecological niches having prospective sources of natural bioactive agents for diverse pharmaceutical, agriculture and industrial applications and should be renewable, eco-friendly and easily obtainable [89]. The discovery of novel bioactive molecules played major role in the search for new drugs and are the most potent sources for the innovation of novel bioactive molecules. The most prominent producers of natural products (compounds derived from living organisms) can originate within different groups of organisms including plants and microorganisms (fungi, bacteria, and actinomycetes) [90]. The techniques involved in the discovery of natural products are isolation, structural elucidation and establishing the bio-synthetic pathway of the secondary metabolites. The area is of substantial interest to scientists due to the structural diversity, complexity and various bioactivities of isolated compounds [91] such as, bio-prospecting of endophytes from medicinal plants is one of the techniques to obtain the bioactive potential metabolites for the preparation of new medicines. Bio-prospecting of endophytes from medicinal plants will be the innovative method for drug discovery which has least environmental consequences and can play significant role in conservation of critically endangered medicinal plants. 

## 13. Conclusions

The review highlights the important vital phytochemicals of the medicinal plant *G. kurroo* Royle, and various experiments have shown scientifically the potential of antibacterial, antioxidant, anti-arthritic, anti-inflammatory, analgesic activities and anti-diabetic activity. The outcome of the study validates the folkloric use of the medicinal plant; however, the antifungal activity of the same plant is yet to be confirmed. The traditional extraction of medicinal plants on a large scale could be minimized by adopting modern techniques, called the bio-prospecting of endophytes, from the medicinal plant for the production and characterization of bioactive metabolites for pharmacological uses that may show a broad spectrum of pharmacological activities and can become a conservative measure for the critically endangered medicinal plant.

## Figures and Tables

**Table 1:** Phytoconstituents of *G. kurroo* Royle.

Root and Rhizome	References
Tannins Alkaloids Saponins Glycosides (Gentiopicrine, Gentianine) Terpenes Flavonoids Phenolics Carbohydrates Genianic Acid Pectin	[29]
	[50]
	[46]
	[36]
	[47]
	[55]
	[48]
	[56]
	[57]
	[58]
	[59]
	[60]
**Flower Tops**	
Alkaloids	[61] [62] [63]
Flavonoids ( Robinetin-0, Luteolin, Apigenin, Kaempferol, Kaempferid)	
Glycosides	
Free Phenols	
Terpense/Sterols	
**Leaves**	
Iridoid Glycoside	[53] [51] [52]
2′-(2,3-Dihydroxybezoyloxy)-7-Ketologanin	
Volatile Aroma Compounds	
Dimethyl Sulphide	
2-Ethylfuran	
1,8-Cincole	
Α-Terpinyl Acetate	
Methandriol	
1,3-Propanediol	
2-Methyl Sulphide	
3-Methyl Butanol	
Pentanol	
Hexanal	
7-Oxabicylo(4,1,0)-Heptanes	

**Table 2:** Quantitative estimation (%) of chemical components and name of fraction of Flavonoids of flower tops of *G. kurroo* Royle.

Flower Tops	%	Name of Flavonoid	Reference
Phenols	2.91 ± 0.07	Robinetin-0	[63]
Alkaloids	0.33 ± 0.02	Luteolin	
Sterols/Terpenes	1.35 ± 0.01	Apigenin	
Flavonoids	0.31 ± 0.01	Kaempferol & Kaempferid	

**Table 3:** Total flavonoid (aluminium chloride colorimetric method by [64] and phenolic content (Folin–Ciocalteu reagent method by [65] of root and leaf extracts of *G. kurroo* Royle.

Extracts	Total Flavonoid Content ^b^	Total Phenolic Content ^a^	Reference
Leaf extract	20 ± 1.5	34 ± 1.8	[59]
Root extract	41 ± 2.2	68 ± 2.4	

Each value is a mean of three biological replicas. ^a^ mg gallic acid equivalent (GAE)/g DW; ^b^ mg rutin equivalent/g DW.

**Table 4:** Phytochemical screening of methanolic extract of *G. kurroo* Royle root.

Phytoconstituents	Test	Reference
Flavonoids	++	[58]
Tannins	++	
Phenolics	++	
Alkaloids	+	
Saponins	+	
Cardiac glycosides	++	
Terpenes	++	
Carbohydrates	+	

++: strong presence; +: moderate presence.

**Table 5:** Antimicrobial activity of *G. kurroo* extracts (minimal inhibitory concentration (MIC) value expressed in mg/mL).

Microorganism	Leaf Extract	Root Extract	Streptomycin	Source
*Proteus mirabilis*	0.27 ± 0.01	0.24 ± 0.04	0.055 ± 0.002	[59]
*Streptococcus faecalis*	0.29 ± 0.02	0.22 ± 0.04	0.025 ± 0.002	
*Escherichia coli*	0.75 ± 0.01	0.67 ± 0.06	0.055 ± 0.001	
*Salmonella enteritidis*	0.90 ± 0.02	0.75 ± 0.05	0.020 ± 0.003	
*Micrococcus luteus*	0.22 ± 0.08	0.15 ± 0.04	0.020 ± 0.004	
*Enterobacter cloacae*	0.60 ± 0.04	0.55 ± 0.03	0.015 ± 0.001	

Each value is a mean of three biological replicas.

**Table 6:** The DPPH scavenging and superoxide scavenging activity determined by DPPH [82] and NBT assay [83].

Extracts (600 μg/mL)	DPPH Assay (%)	NBT Assay (%)	Source
Leaf	53	51	[59]
Root	72	63	
**Control**			
Vitamin C/Ascorbic acid *	91	91.7	

DPPH (1,1-Diphenyl-2-picrylhydrazyl) & NBT(nitroblue tetrazolium); * Ascorbic acid taken as a positive control.
